# 4-Hydroxychalcone Induces Cell Death via Oxidative Stress in *MYCN*-Amplified Human Neuroblastoma Cells

**DOI:** 10.1155/2019/1670759

**Published:** 2019-12-05

**Authors:** Amnah M. Alshangiti, Eszter Tuboly, Shane V. Hegarty, Cathal M. McCarthy, Aideen M. Sullivan, Gerard W. O'Keeffe

**Affiliations:** ^1^Department of Anatomy and Neuroscience, University College Cork, Cork, Ireland; ^2^Cork Neuroscience Centre, University College Cork, Cork, Ireland; ^3^Department of Pharmacology and Therapeutics, University College Cork, Cork, Ireland

## Abstract

Neuroblastoma is an embryonal malignancy that arises from cells of sympathoadrenal lineage during the development of the nervous system. It is the most common pediatric extracranial solid tumor and is responsible for 15% of childhood deaths from cancer. Fifty percent of cases are diagnosed as high-risk metastatic disease with a low overall 5-year survival rate. More than half of patients experience disease recurrence that can be refractory to treatment. Amplification of the *MYCN* gene is an important prognostic indicator that is associated with rapid disease progression and a poor prognosis, highlighting the need for new therapeutic approaches. In recent years, there has been an increasing focus on identifying anticancer properties of naturally occurring chalcones, which are secondary metabolites with variable phenolic structures. Here, we report that 4-hydroxychalcone is a potent cytotoxin for *MYCN*-amplified IMR-32 and SK-N-BE (2) neuroblastoma cells, when compared to non-*MYCN*-amplified SH-SY5Y neuroblastoma cells and to the non-neuroblastoma human embryonic kidney cell line, HEK293t. Moreover, 4-hydroxychalcone treatment significantly decreased cellular levels of the antioxidant glutathione and increased cellular reactive oxygen species. In addition, 4-hydroxychalcone treatment led to impairments in mitochondrial respiratory function, compared to controls. In support of this, the cytotoxic effect of 4-hydroxychalcone was prevented by co-treatment with either the antioxidant N-acetyl-L-cysteine, a pharmacological inhibitor of oxidative stress-induced cell death (IM-54) or the mitochondrial reactive oxygen species scavenger, Mito-TEMPO. When combined with the anticancer drugs cisplatin or doxorubicin, 4-hydroxychalcone led to greater reductions in cell viability than was induced by either anti-cancer agent alone. In summary, this study identifies a cytotoxic effect of 4-hydroxychalcone in *MYCN*-amplified human neuroblastoma cells, which rationalizes its further study in the development of new therapies for pediatric neuroblastoma.

## 1. Introduction

Neuroblastoma (NB) is the most common extracranial solid pediatric cancer and the most frequent cancer diagnosed in the first year of life [1]. The incidence of NB is 10-20 cases per million children [1, 2], yet NB is responsible for 15% of childhood deaths from cancer [3, 4]. NB is an embryonal malignancy that arises from cells of the sympathoadrenal lineage and can arise anywhere in the developing sympathetic nervous system [5–7]. Amplification of the *MYCN* gene is an important prognostic indicator that is associated with rapid disease progression and poor prognosis, irrespective of patient age or disease stage [8–10]. *MYCN* is amplified in about 20% of NB cases, and these tumors display an undifferentiated and aggressive phenotype [11, 12]. Moreover, in high-risk NB without *MYCN* amplification, there is often high Myc pathway activity, highlighting the importance of Myc as a driver of high-risk metastatic disease [13]. Indeed, *MYCN* amplification has been associated with the lowest response rate of NB after chemotherapy [14]. Half of affected children are diagnosed with high-risk metastatic disease, and despite intensive multimodal therapy [15, 16], the overall 5-year survival rate is just 40-50% [16]. Furthermore, over half of patients experience disease recurrence, and this can be refractory to treatment [9, 17]. There is therefore a continuing need to identify new compounds that are potential cytotoxins for high-risk, *MYCN*-amplified NB cells.

While the molecular basis of chemoresistance in *MYCN*-amplified NB cells is multifactorial, the resistance of *MYCN*-amplified human NB cells to oxidative stress through transcriptional upregulation of glutamate cysteine ligase (GCL) plays an important role [18]. GCL is the rate-limiting enzyme in the production of the most abundant cellular antioxidant, glutathione (GSH). GSH is a target for chemotherapeutic reagents, and it is elevated in cancer cells, at levels which correlate with metastatic potential and with drug resistance [19, 20]. GSH has also been targeted for the induction of cell death in NB cells, through both *MYCN* and P53 [21]. For this reason, compounds that induce oxidative stress or that deplete GSH levels may have promising potential as therapies for NB.

In recent years, there has been increasing interest in the anti-cancer effects of naturally occurring compounds [22–25]. One group of compounds that has received significant attention is the flavonoids, which is a broad class of secondary metabolites with variable phenolic structures [26]. Chalcones are a subclass of flavonoids that have an open-chain structure in which two aromatic rings, known as the A and B rings, are joined by a three-carbon *α*, *β*-unsaturated carbonyl system [27, 28]. As the chalcone structure can be modified with relative ease [29, 30], and since chalcones have been reported to be the most potent flavonoid subclass in terms of antitumour activity [29, 31, 32], there has been significant interest in chalcone-based anticancer drug development [33, 34]. Previous reports suggest that the anticancer effects of chalcones are associated with the presence of hydroxyl groups, and consequently, hydroxylated chalcone derivatives have the greatest anticancer effects [31, 32, 35]. The position of the hydroxyl group is important in regulating the biological activity of chalcones [36, 37]. Hydroxychalcones have been reported to deplete GSH levels in hepatocytes [38], indicating that they may have chemotherapeutic potential. A study that compared the cytotoxic effects of thirteen hydroxychalcones on melanoma cells confirmed that the number of hydroxyl groups in the molecule affects the strength of their cytotoxic activity [39]. Our previous work found that the chalcone isoliquiritigenin (ISLQ) had cytotoxic effects on NB cells, which were induced through elevation of reactive oxygen species (ROS), leading to activation of necroptotic cell death [40].

Structurally, 4-hydroxychalcone (4HC) is perhaps the simplest hydroxylated chalcone, and it has been shown to have an antiangiogenic effect that may be useful in halting cancer spread [41]. There is some evidence for cytotoxic effects of other hydroxylated chalcones in melanoma [39], colon [42], glioma [43], breast [44], and leukemia [45] cancer cells. In addition, 4′-hydroxychalcone, an isomer of 4HC, has been reported to induce cytotoxic effects, via mitochondrial dysfunction, in human SH-SY5Y NB cells [46]. However, to our knowledge, no studies have examined the direct anticancer effects of 4HC in *MYCN*-amplified NB cells. In the present study, we investigated whether 4HC is cytotoxic to *MYCN*-amplified NB cells and we studied its mode of cell death, its effects on oxidative stress, and its effects when combined with the anticancer drugs, cisplatin and doxorubicin.

## 2. Materials and Methods

### 2.1. Culture of Cell Lines

SK-N-BE (2) cells (ATCC) were cultured in Minimum Essential Medium supplemented with 100 nM L-glutamine, 1% penicillin-streptomycin, 1% 1 : 1 nonessential amino acid solution: Ham's F-12, and 15% fetal bovine serum (FBS). IMR-32 cells (ATCC) were cultured in Minimum Essential Medium supplemented with 100 nM L-glutamine, 1% penicillin-streptomycin, 1% nonessential amino acid solution, and 10% FBS. SH-SY5Y cells (ATCC) were cultured in Dulbecco's modified Eagle's medium (DMEM)/Nutrient Mixture F-12 Ham's supplemented with 100 nM L-glutamine, 1% penicillin-streptomycin, and 10% FBS. HEK293T cells were cultured in high-glucose DMEM supplemented with 100 nM L-glutamine, 1% penicillin-streptomycin, and 10% FBS (all from Sigma). All cells were cultured at 37°C in a humidified atmosphere with 5% CO_2_.

### 2.2. Primary Culture of E14 Rat VM

Embryonic day (E) 14 rat embryos were removed by laparotomy from time-mated pregnant Sprague-Dawley rats after terminal anesthesia followed by decapitation. Embryos were removed and kept on ice in cold (4°C) Hank's balanced salt solution (HBSS). The ventral midbrain (VM) was microdissected from each embryo and transferred to a tube containing HBSS at 4°C. HBSS was removed, and the tissue pieces were incubated in 0.1% trypsin solution for 20 min at 37°C. Trypsin was inactivated by the addition of FBS, and the tissue pieces were pelleted by centrifugation at 1,100 rpm for 5 min, after which the trypsin/FBS supernatant was discarded. The tissue pieces were resuspended in culture medium consisting DMEM/F12, 33 mM D-glucose, 1% L-glutamine, and 1% FBS (all from Sigma) supplemented with 2% B27 (Invitrogen). Tissue pieces were dissociated to a single cell suspension. Cells were plated on poly-D-lysine-coated (Sigma) 24-well tissue culture plates at a density of 1 x 10^5^ of cells per well in 500 *μ*l of differentiation medium and cultured at 37°C in a humidified atmosphere with 5% CO_2_.

### 2.3. Pharmacological Treatments

Cultures were treated with 4HC (Sigma) (0 to 100 *μ*M) or a vehicle control (methanol (MeOH); Sigma), with or without cisplatin (CP) or doxorubicin (Dox) where indicated. Cells were pretreated for 1 h with 3 *μ*M IM-54 (Calbiochem), or 150 *μ*M Mito-TEMPO, or with N-acetyl-L-cysteine (NAC; Sigma; 300 *μ*M for IMR-32, 1 mM for SK-N-BE (2)), prior to the addition of 4HC. All of these concentrations were empirically determined prior to their use (data not shown).

### 2.4. Cell Viability and Cell Death Assays

Cell viability was determined at 24 h using an 3-(4,5-dimethylthiazol-2-yl)-2,5-diphenyltetrazolium bromide (MTT) assay. At the end of each experiment, cultures were incubated in 1 mg/ml MTT solution for 3.5 h at 37°C. MTT solution was removed, and the crystals were dissolved in DMSO. Spectrophotometric absorbance was measured at 595 nm (with a reference wavelength of 620 nm), and raw absorbances were expressed as a percentage of the control. Cell death was measured using a lactate dehydrogenase (LDH) assay. For this assay, 150 *μ*L of culture medium was removed and centrifuged at 1000 rpm for 5 min to remove cellular debris. LDH activity was measured using an LDH assay kit (ThermoFisher), according to the manufacturer's instructions. Phase-contrast images were captured using an Olympus IX71 inverted microscope fitted with an Olympus DP70 camera.

### 2.5. Measurement of Cellular Reactive Oxygen Species (ROS)

CellRox®, a cell-permeable oxidative stress indicator dye (ThermoFisher), was added to culture medium at a final concentration of 5 *μ*M for 30 min at 37°C. For time-course and dose-response experiments, SK-N-BE (2) were plated and treated with 25, 50, or 100 *μ*M of 4HC for 2, 4, or 6 h, before 5 *μ*M CellRox was added to the medium. For somal and nuclear area measurements, cells were plated and treated for 6 h with 4HC at 10 *μ*M for IMR-32 and 25 *μ*M for SK-N-BE (2), before being labelled with the vital fluorescent dye Calcein-AM (1 : 500; Invitrogen) and the nuclear stain Hoechst (1 *μ*g/ml) then incubated at 37°C for 30 min. Four random, nonoverlapping fields were captured per well in each experiment (in three wells per group for each *n*) using an Olympus IX71 inverted microscope fitted with an Olympus DP70 camera. CellRox fluorescence intensity, somal area, and nuclear size were measured in a blinded fashion using ImageJ in ten random individual cells per field for each well of each group. The intensity/area results were normalized to the control and expressed as a percentage of the control group.

### 2.6. GSH Recycling Assay

GSH levels were measured using a spectrophotometric assay involving oxidation of GSH to glutathione disulphide (GSSG) by the sulfhydryl reagent 5,5′-dithio-bis (2-nitrobenzoic acid) (DTNB) to form the yellow derivative 5′-thio-2-nitrobenzoic acid (TNB). The GSSG formed can be recycled to GSH by glutathione reductase in the presence of *β*-NADPH [47]. SK-N-BE (2) and IMR-32 were plated in 6-well plates at the density of 10^6^ per well for 72 h and then treated with 25 or 50 *μ*M 4HC for 4 h. At the end of the incubation time, cells and medium were removed by mechanical scraping using a sterilized rubber cell scraper in 1.5 ml Eppendorf tubes and centrifuged for 5 min at 1000*g* at 4°C. The media was discarded and the pellet was washed in 0.5 ml cold PBS before being centrifuged again. The supernatant was removed and the pellet was resuspended in extraction buffer (0.1% Triton X-100 and 0.6% sulfosalicylic acid in 0.1 M potassium phosphate buffer with 5 mM EDTA disodium salt, pH 7.5 (KPE buffer). The cells were sonicated and vortexed repeatedly, before two cycles of freezing and defrosting to ensure complete cell lysis. Cell lysates were centrifuged for 4 min at 3000*g* at 4°C, then the supernatant was removed. 20 *μ*l of those supernatant samples, or GSH standards, was added into 96-well plate with 120 *μ*l of 1 : 1 DTNB to glutathione reductase. 60 *μ*l of *β*-NADPH was added, and absorbance measured at a wavelength of 405 nm at 0, 30, 60, 90, and 120 s time points. Total GSH was calculated from the linear regression obtained from the standard curve of each time point, as previously described [47]. All samples were normalized for total amounts of protein using a BCA Bradford assay kit according to the manufacturer's instructions (ThermoFisher).

### 2.7. Analysis of Mitochondrial Function Using Agilent Seahorse XF 96

For analysis of mitochondrial function, IMR-32 and SK-N-BE (2) were plated in 96-well plates at 4 × 10^5^ cells per well. On reaching 90% confluence, cells were treated with 50 *μ*M 4HC for 4 h before mitochondrial respiration was measured using the Seahorse XF 96 Extracellular Flux Analyser and XF Wave Analysis software version 1.4. (Agilent Technologies, Santa Clara, CA, USA) and Seahorse XF 96 Extracellular Flux assay kits. All chemicals were purchased from Agilent Technologies (Santa Clara, USA). Following the 4 h drug treatment, cell culture media was replaced with 200 *μ*l/well DMEM-based XF modified media (free of bicarbonate and phenol red, containing 25 mM glucose and 1 mM pyruvate), and cell plates were incubated for 60 min in a non-CO_2_ incubator before sensor calibration was performed and mitochondrial respiration stress test experiments were initiated at a consistent temperature of 37°C. The instrument was programmed for 3 cycles of drug injection, followed by 3 mixing steps and 3 measuring periods (3 min each). The overall experimental time was 110 min. All oxygen consumption rate (OCR) analyses were carried out at least 3 times, with a minimal of 6-12 technical replicates for each treatment. Four different compounds were used to assess mitochondrial OCR: 1.5 *μ*M oligomycin, an inhibitor of mitochondrial membrane adenosine triphosphate (ATP) synthase (F1F0 ATP synthase); 1.0 *μ*M carbonilcyanide p-triflouromethoxyphenylhydrazone (FCCP), an ionophore uncoupler of oxidative phosphorylation; 0.3 *μ*M antimycin A, which inhibits the mitochondrial electron transport chain from cytochrome b to cytochrome C1; and 1.0 *μ*M rotenone, an inhibitor of mitochondrial electron transport at nicotinamide adenine dinucleotide: ubiquinone oxidoreductase in complex I.

### 2.8. Statistical Analysis

To analyze significant differences, *T* test, or one-way ANOVA with *post hoc* Fisher's or Tukey's test, was carried out where indicated. All data are presented as mean ± SEM, and all experiments were repeated at least three times. Differences were deemed significant when *p* < 0.05.

## 3. Results

### 3.1. 4HC Has Potent Cytotoxic Effects on Several MYCN-Amplified and Non-MYCN-Amplified Cell Lines

**Figure 1:**
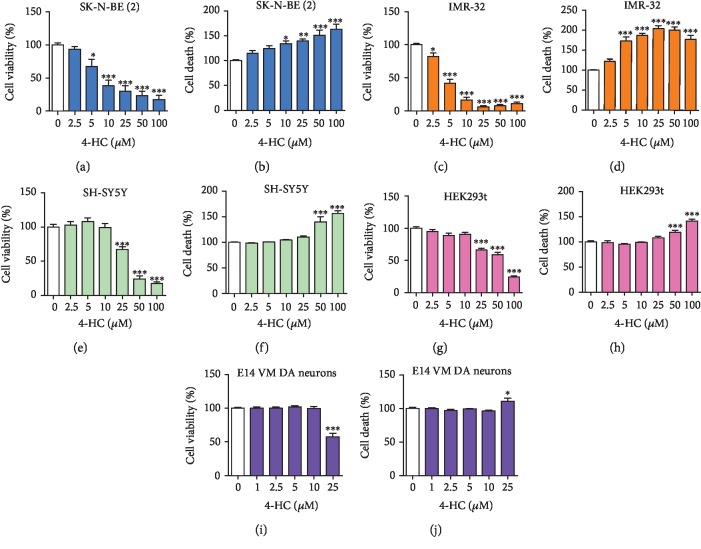
4HC has potent cytotoxic effects on several *MYCN*-amplified and non-*MYCN*-amplified cell lines. Cell viability measured by MTT assay (a, c, e, g, i) and cell death measured by LDH assay (b, d, f, h. j) on (a, b) SK-N-BE (2), (c, d) IMR-32, (e, f) SH-SY5Y, (g, h) HEK293t cells, and (i, j) primary cultures of E14 rat midbrain, treated with the indicated concentrations 4HC for 24 h or 3DIV for E14 cells. All data are mean ± SEM; *n* = 3 independent experiments for A-H, *n* = 6 independent experiments for (i) and (j); ^∗^*p* < 0.05, ^∗∗^*p* < 0.01, and ^∗∗∗^*p* < 0.001 versus control; one-way ANOVA with Tukey's test.

We first sought to determine whether 4HC had cytotoxic effects on *MYCN*-amplified NB cells. SK-N-BE (2) and IMR-32 cells were treated for 24 h with increasing concentrations of 4HC, ranging from 0 to 100 *μ*M. MTT and LDH assays were used to assess cell viability and cell death. These analyses revealed a concentration-dependent effect of 4HC on NB cell viability (Figure 1). Specifically, MTT assays showed that treatment with ≥5 *μ*M 4HC in SK-N-BE (2) cells (Figure 1(a)), or ≥2.5 *μ*M 4HC in IMR-32 cells (Figure 1(c)), led to a significant reduction in cell viability. SK-N-BE (2) and IMR-32 cells treated with the equivalent amount of vehicle control (MeOH) displayed no significant reductions in cell viability (Supplementary Figures [Supplementary-material supplementary-material-1] and [Supplementary-material supplementary-material-1]), indicating a cytotoxic effect of 4HC on *MYCN*-amplified NB cells. To confirm these findings, LDH assays were used and revealed increases in LDH activity in both IMR-32 and SK-N-BE (2) cells after 4HC treatment, which complemented the results of the MTT assays. Specifically, increasing concentrations of 4HC led to significant increases in LDH activity in both SK-N-BE (2) cells (Figure 1(b)) and IMR-32 cells (Figure 1(d)). To determine if this was a general cytotoxic effect of 4HC, we examined the effects of 4HC on SH-SY5Y cells, a non-*MYCN*-amplified NB cell line and on a non-NB human embryonic kidney cell line, HEK293t. MTT and LDH assays revealed that 4HC treatment affected both SH-SY5Y (Figures 1(e) and 1(f)) and HEK293t (Figure 1(g) and 1(h)) cell viability only at concentrations of ≥25 *μ*M. SH-SY5Y and HEK293t cells treated with the vehicle control (MeOH) displayed no significant reductions in cell viability (Supplementary Figures [Supplementary-material supplementary-material-1] and [Supplementary-material supplementary-material-1]). To further confirm this, we examined the effects of 4HC on primary cultures of E14 rat midbrain and found that 4HC treatment for 3 DIV was not toxic to these cells at concentrations of less than 25 *μ*M (Figures 1(i) and 1(j)). Comparison of the effects of 4HC treatment on cell viability in SK-N-BE (2) and IMR-32 cells with our previous study on the chalcone ISLQ in these cell lines [40] showed that 10 *μ*M 4HC treatment for 24 h induced a significantly greater reduction in cell viability than the same dose of ISLQ, in both of these cell types (Supplementary Figures [Supplementary-material supplementary-material-1]). This suggests that MYCN-amplified NB cells have differential sensitivity to distinct chalcones.

### 3.2. MYCN-Amplified NB Cells Are more Sensitive to the Effects of 4HC than Non-MYCN-Amplified Cell Lines

**Figure 2:**
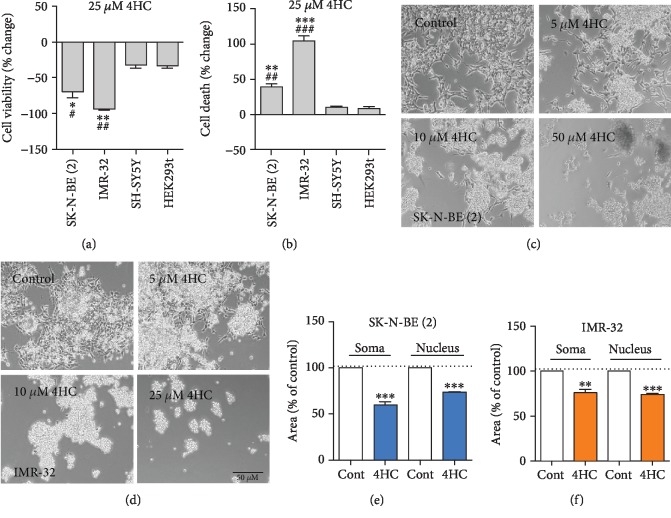
*MYCN*-amplified NB cells are more sensitive to the effects of 4HC than non-*MYCN*-amplified cell lines. Percentage change in (a) cell viability measured by MTT assay and (b) cell death measured by LDH assay on the four indicated cell lines after treatment with 25 *μ*M 4HC for 24 h. All data are mean ± SEM; *n* = 3 independent experiments. ^∗^*p* < 0.05, ^∗∗^*p* < 0.01, and ^∗∗∗^*p* < 0.01 compared to SH-SY5Y cells and ^#^*p* < 0.05, ^##^*p* < 0.01, and ^###^*p* < 0.001 compared to HEK283t cells; one-way ANOVA with Fisher's LSD test. Representative phase contrast micrographs of (c) SK-N-BE (2) cells and (d) IMR-32 cells treated with the indicated concentrations of 4HC for 24 h. Scale bar = 50 *μ*m. Somal and nuclear areas as percentages of controls in (e) SK-N-BE (2) cells treated with 25 *μ*M 4HC for 6 h or (f) IMR-32 treated with 10 *μ*M 4HC for 6 h. All data are mean ± SEM; *n* = 3 independent experiments. ^∗∗^*p* < 0.01 and ^∗∗∗^*p* < 0.01 compared to controls (Cont) for each parameter; Students *t* test for each parameter in each cell type.

To specifically examine the sensitivity of *MYCN*-amplified NB cells, we compared the percentage changes on the MTT and LDH assays in each of the four cell lines after treatment with 25 *μ*M 4HC. This showed that 4HC had a significantly greater cytotoxic effect on SK-N-BE (2) and IMR-32 cells than on SH-SY5Y and HEK293t cells (Figures 2(a) and 2(b)). From this point on, we focused on studying the role of 4HC in the *MYCN*-amplified NB cell lines, SK-N-BE (2), and IMR-32.

To determine whether 4HC treatment led to morphological changes consistent with cell death, we examined cell morphology in SK-N-BE (2) (Figure 2(c)) and IMR-32 (Figure 2(d)) cells, using Calcein-AM and Hoechst staining. Significant reductions in both cell area and nuclear area were induced by treatment with 25 *μ*M 4HC in SK-N-BE (2) cells (Figure 2(e)) and 10 *μ*M 4HC in IMR-32 cells for 6 h (Figure 2(f)). Collectively, these data show that 4HC can induce cell shrinkage and are consistent with the data showing reductions in cell viability and increases in cell death.

### 3.3. 4HC Depletes the Antioxidant Glutathione and Increases Oxidative Stress in NB Cells

**Figure 3:**
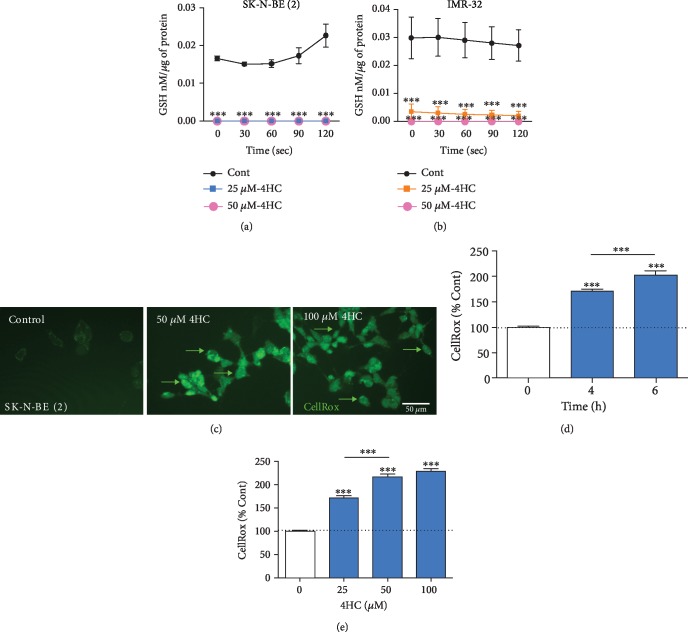
4HC depletes the antioxidant glutathione and increases oxidative stress in NB cells. GSH concentration in (a) SK-N-BE (2) cells and (b) IMR-32 cells treated with 50 and 25 *μ*M 4HC for 4 h. All data are mean ± SEM; *n* = 3 independent experiments. ^∗^*p* < 0.05 and ^∗∗∗^*p* < 0.01 versus control (Cont) at each time point; two-way ANOVA with Sidak's post hoc test. (c) Representative photomicrographs of CellRox fluorescence intensity in SK-N-BE (2) cells treated 50 or 100 4HC for 6 h. Arrows indicate elevated ROS levels in individual cells. Scale bar = 50 *μ*m. CellRox fluorescence intensity as percentages of control in SK-N-BE (2) cells treated with (d) 25 *μ*M 4HC for 2, 4, or 6 h and (e) 25, 50, or 100 *μ*M 4HC for 2 h. All data are mean ± SEM of 360 cells from *n* = 3 independent experiments. ^∗∗∗^*p* < 0.001 versus control; ANOVA with Fisher's LSD test.

We next examined the pathways involved in 4HC-induced cell death in NB cells. Chalcones have been shown to regulate cellular levels of ROS, depending on the cellular type and context [48]. Chalcones also have been reported to induce depletion of the antioxidant GSH, which is considered a potential target for cancer treatment [49]. We first examined whether 4HC treatment induced oxidative stress in *MYCN*-amplified NB cells. We used the enzymatic recycling method of measuring the reduced GSH form of the antioxidant glutathione [47]. GSH is tripeptide thiol (*γ*-glutamyl cysteinyl glycine) cellular antioxidant that acts as a free radical scavenger, and its intracellular concentration is an indicator of oxidative stress. In cells undergoing oxidative stress, there is a reduction in cellular GSH levels. Cells were treated with higher doses of 4HC for a shorter time, to examine the early effect of 4HC treatment on ROS levels in living cells. SK-N-BE (2) and IMR-32 cells were treated with 25 or 50 *μ*M 4HC for 4 h before GSH levels were measured. 4HC treatment led to significantly lower GSH levels in both SK-N-BE (2) cells (Figure 3(a)) and IMR-32 cells (Figure 3(b)). To gather further evidence to support that finding that 4HC induces oxidative stress, we loaded SK-N-BE (2) cells with CellRox, which is a compound that emits fluorescence with an intensity that correlates with intracellular levels of ROS, a measure of cellular oxidative stress (Figure 3(c)). Treatment with 25 *μ*M 4HC led to a significant increase in cellular ROS after 4 and 6 h (Figure 3(d)). Treatment with 25 *μ*M, 50 *μ*M, or 100 *μ*M 4HC for 2 h induced a concentration-dependent increase in cellular ROS (Figure 3(e)). Collectively, these data show that 4HC increases cellular oxidative stress in SK-N-BE (2) and IMR-32 cells.

### 3.4. 4HC-Induced Cell Death Affects Oxygen Consumption Rate in NB Cells

**Figure 4:**
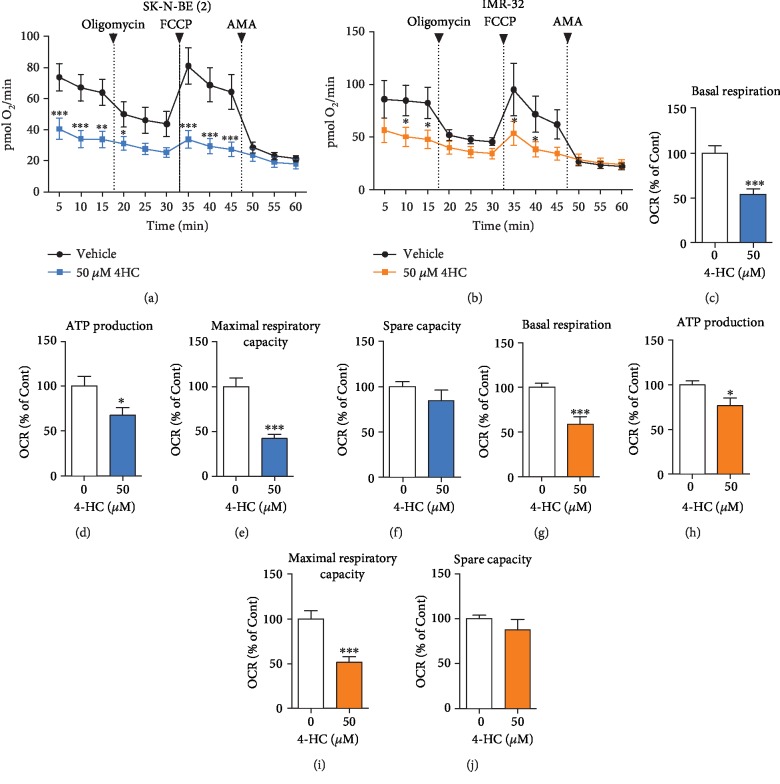
4HC-induced cell death affects oxygen consumption rate in NB cells. Mitochondrial oxygen consumption rate (OCR) of (a) SK-N-BE(2) and (b) IMR-32 cells treated with 50 *μ*M 4HC or vehicle (MeOH) for 4 h, at various stages: initially, “basal respiration” cellular OCR; then, OCR after treatment with oligomycin, a complex V inhibitor (“ATP production”), then OCR after treatment with FCCP, a protonophore (“maximal respiratory capacity”), and finally, OCR after treatment with AMA (“spare capacity”. OCR at each of the four individual stages in (c-f) SK-N-BE (2) and (g–j) IMR-32 cells from the same experiment, presented as a percentage of vehicle. All data are mean ± SEM, *n* = 3 independent experiments. ^∗^*p* < 0.05, ^∗∗^*p* < 0.01, and ^∗∗∗^*p* < 0.001 versus untreated; ANOVA with Fisher's LSD test, unpaired *T* test.

To further investigate cell death induced by 4HC in NB cells, we performed an analysis of bioenergetic state by measuring the rate of oxygen consumption in cells treated with 50 *μ*M 4HC for 4 h, using the Seahorse XF 96 Extracellular Flux Analyser. 4HC-treated cells showed impairment in respiration function throughout the experiment, compared to the vehicle (Figures 4(a) and 4(b)). Specifically, there was a significant decrease in basal respiration levels in both IMR-32 and SK-N-BE (2) cells after treatment with 4HC (Figures 4(c) and 4(g)). Addition of oligomycin, an inhibitor of mitochondrial ATP-synthase, caused a decrease in ATP production. Consistent with the changes in basal respiration, there was also a reduction in mitochondrial ATP synthase in 4HC-treated cells (Figures 4(d) and 4(h)). Subsequent addition of FCCP caused uncoupling of mitochondrial oxidative phosphorylation (OX-PHOS) to induce maximal respiration. Treatment with 4HC led to a significant decrease in maximal respiration capacity, indicative of uncoupling (Figures 4(e) and 4(h)). Finally, a combination of rotenone and antimycin A was added to inhibit complex I of the mitochondrial respiration chain. Following these treatments, there was no notable effect of 4HC on the spare capacity of either of the two cell lines (Figures 4(f) and 4(j)).

### 3.5. 4HC-Induced NB Cell Death Is Prevented by Inhibition of Oxidative Stress and by Scavenging of Mitochondrial Superoxide

**Figure 5:**
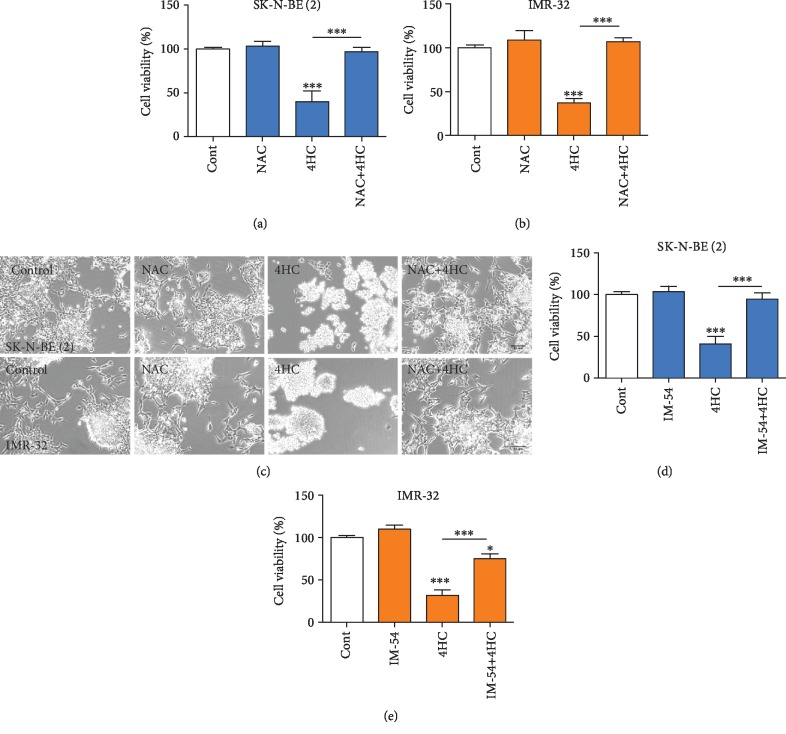
4HC-induced NB cell death is prevented by inhibition of oxidative stress. (a, b) Cell viability measured by MTT assay and (c) representative photomicrographs of (a, c) SK-N-BE (2) and (b, c) IMR-32 cells pretreated with NAC before being cultured with or without 25 *μ*M 4H for SK-N-BE (2) and 10 *μ*M 4H for IMR-32 C for 24 h. Scale bar = 50 *μ*m. Cell viability measured by MTT assay on (d) SK-N-BE (2) and (E) IMR-32 cells pretreated with IM-54 before being cultured with or without 25 *μ*M 4HC for SK-N-BE (2) and 10 *μ*M 4HC for IMR-32 for 24 h. All data are mean ± SEM; *n* = 3 independent experiments. ^∗^*p* < 0.05 and ^∗∗∗^*p* < 0.001 versus control or as indicated; ANOVA with Fisher's LSD test).

To determine whether increases in oxidative stress were involved in 4HC-induced cell death, we used two complementary approaches. Firstly, SK-N-BE (2) and IMR-32 cells were pretreated for 2 h with the antioxidant NAC prior to the addition of 4HC at 25 *μ*M for SK-N-BE (2) and 10 *μ*M for IMR-32, before cell viability was examined after 24 h using an MTT assay. These analyses revealed that 4HC treatment led to a significant reduction in cell viability in both SK-N-BE (2) (Figure 5(a)) and IMR-32 cells (Figure 5(b)), which was fully prevented by NAC pretreatment in both cell lines (Figures 5(a)–5(c)).

**Figure 6:**
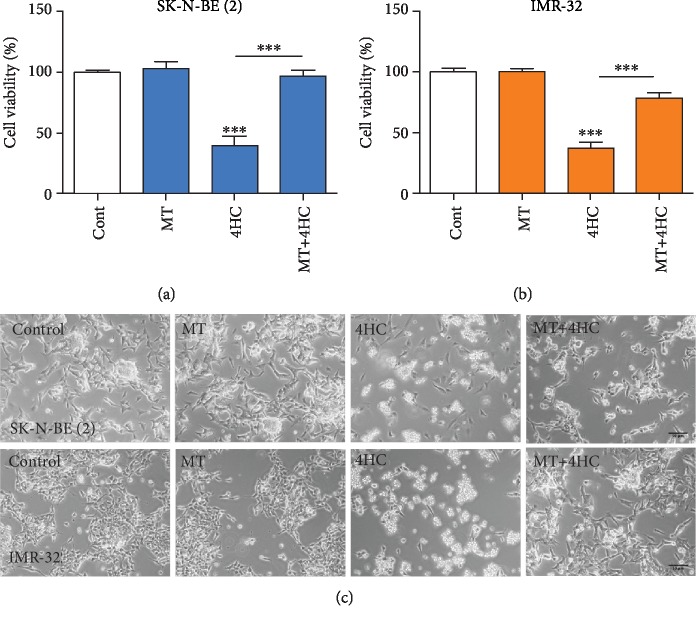
4HC-induced NB cell death is prevented by scavenging of mitochondrial superoxide. (a, b) Cell viability measured by MTT assay and (c) representative photomicrographs of (a, c) SK-N-BE (2) and (b, c) IMR-32 cells pretreated with mito-TEMPO (MT) before being cultured with or without 25 *μ*M of 4HC or SK-N-BE (2) and 10 *μ*M 4HC for IMR-32 for 24 h. Scale bar = 50 *μ*m. All data are mean ± SEM; *n* = 3 independent experiments. ^∗∗∗^*p* < 0.001 versus control or as indicated; ANOVA with Fisher's LSD test.

To confirm these findings, we examined whether a small molecule inhibitor (IM-54), which selectively blocks oxidative stress-induced cell death [50], could prevent the cell death induced by 4HC. To do this, SK-N-BE (2) cells and IMR-32 were pretreated for 1 h with 3 *μ*M IM-54, before 4HC was added for 24 h. In agreement with the NAC experiment, 4HC treatment for 24 h led to a significant reduction in cell viability in SK-N-BE (2) (Figure 5(d)) and in IMR-32 cells (Figure 5(e)), which was fully prevented by IM-54 pretreatment in both cell lines. In agreement with this, IM-54 prevented the increase in cellular ROS following 4HC treatment in SK-N-BE (2) cells (Supplementary [Supplementary-material supplementary-material-1]). To further study the role of mitochondrial ROS in 4HC-induced cell death, we pretreated the cells with Mito-TEMPO, a compound that scavenges superoxide by mimicking superoxide dismutase from the mitochondria [51]. Mito-TEMPO significantly protected SK-N-BE (2) and IMR-32 cells against 4HC-induced cytotoxicity (Figure 6). Collectively, these data show that 4HC-induced oxidative stress plays a functional role in 4HC-induced cell death in SK-N-BE (2) and IMR-32 NB cells.

### 3.6. 4HC Enhances Cytotoxicity Induced by Doxorubicin and Cisplatin in SK-N-BE (2) and IMR-32 Cells

**Figure 7:**
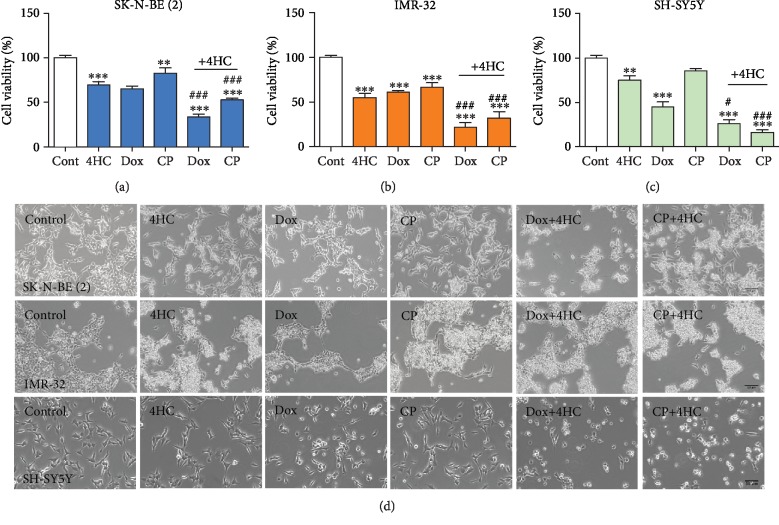
4HC enhances cytotoxicity induced by doxorubicin and cisplatin in SK-N-BE (2) and IMR-32 cells. (a–c) Cell viability measured by MTT assay and (d) representative photomicrographs of (a) SK-N-BE (2) and (b) IMR-32 treated with 5 *μ*M 4HC and (c) SH-SY5Y cells treated with 25 *μ*M 4HC, or with 5 *μ*M doxorubicin (Dox) or 100 *μ*M cisplatin (CP) with or without 4HC for 24 h. All data are mean ± SEM. ^∗^*p* < 0.05, ^∗∗^*p* < 0.01, and ^∗∗∗^*p* < 0.001 versus control. ^###^Combined treatment versus Dox or CP alone; ANOVA with Fisher's LSD test. Scale bar = 50 *μ*m.

Given the toxicity associated with high-dose chemotherapy, it is important to identify agents that enhance the cytotoxic effects of commonly used anticancer drugs such as cisplatin and doxorubicin. To this end, we performed dose-response experiments with doxorubicin in both SK-N-BE (2) and IMR-32 cells and assessed cell viability at 24 h using an MTT assay. Treatment with 10 *μ*M doxorubicin resulted in approximately 50% decrease in cell viability at 24 h in both cell lines (Supplementary [Supplementary-material supplementary-material-1]). Both cell lines, as well as the non-*MYCN*-amplified SH-SY5Y cell line, were treated with 5 *μ*M doxorubicin and 100 *μ*M cisplatin for 24 h, with or without cotreatment with 4HC. The addition of 4HC resulted in a significantly greater cytotoxic effect than treatment with either of the anticancer drugs alone, in all three cell lines, SK-N-BE (2) (Figures 7(a) and 7(d)), IMR-32 (Figures 7(b) and 7(d)), and SH-SY5Y (Figures 7(c) and 7(d)).

## 4. Discussion

The use of chalcones as potential anticancer agents is relatively novel. To date, there have been no reports on the potential of 4HC as an anticancer agent in human *MYCN*-amplified NB. In the current study, we found that 4HC exerted potent cytotoxic effects on *MYCN*-amplified SK-N-BE (2) and IMR-32 human NB cells at low concentrations, in comparison to other non-*MYCN*-amplified NB cells, kidney embryonic cells, or healthy murine primary neuronal cultures. We also found that the cytotoxicity induced in *MYCN-*amplified NB cell lines by 4HC was higher than that induced by ISLQ, another chalcone which we had investigated in a previous study [40]. The cytotoxic effect of 4HC treatment was found to be both time- and concentration-dependent and to be accompanied by decreases in both somal and nuclear area. The mechanism of the cell death induced by 4HC involved elevation of intracellular ROS combined with significant depletion of the antioxidant GSH. Furthermore, we found that replenishing the depleted antioxidants by treatment with NAC, or blocking oxidative stress signalling by treatment with the small molecule IM-54, or protecting the mitochondria using Mito-TEMPO, a mitochondria-specific superoxide scavenger, could significantly protect cells from 4HC-induced cytotoxicity. In addition, 4HC treatment caused a severe impairment of mitochondrial oxygen consumption in both of the *MYCN*-amplified cell lines. Cotreatment with 4HC was also found to increase the cytotoxic effects of cisplatin and doxorubicin on SK-N-BE (2), IMR-32, and SH-SY5Y cells.

In agreement with our current findings, previous studies have documented the anticancer activity of other chalcones on a wide variety of cancer cells. Isobavachalcone, a chalcone constituent of *Angelica keiskei*, was found to induce caspase-dependent cell death in IMR-32 and NB-39 NB cells, when applied at a concentration 10 *μ*M for 24 h [52]. ISLQ and phloretin, similar chalcones to 4HC, have also been found to exert cytotoxic effects on B16 melanoma cells [53], while the dihydrochalcone phloretin can induce cell death via apoptosis in HL60 human leukemia cells [54]. In addition, the cytotoxic effect of the synthetic naphthyl chalcone, (2E)-1-(2,5-dimethoxy-phenyl)-3-(1-naphthyl)-2-propene-1-one, was reported to be both concentration- and time-dependent, with IC50 values ranging between 1.5 *μ*M and 40 *μ*M, in K562, Jurkat, Kasumi, U937, CEM, and NB4 cells [38, 55]. Moreover, another chalcone, *trans*-chalcone, has been shown to suppress the growth of U2OS osteosarcoma cells in a concentration- and time-dependent manner, with significant inhibition observed after treatment for 48 h [56]. Recently, de Moura Escobar and colleagues have shown cytotoxic effects of 10-60 *μ*M 4′-hydroxychalcone, an isomer of 4HC, on SH-SY5Y cells [46]. Collectively, these data are consistent with our current findings, which show that 4HC has cytotoxic effects on *MYCN*-amplified NB cells at concentrations of ≥5 *μ*M in SK-N-BE (2) and ≥2.5 *μ*M in IMR-32 cells. These cytotoxic effects of 4HC on NB cells are also consistent with our previous work on the chalcone-derived flavonoid, ISLQ. In that study, we found that treatment with ≥5 *μ*M ISLQ for 24 h had potent cytotoxic effects on both SK-N-BE (2) and IMR-32 cells [40]. 4HC had a greater cytotoxic effect on *MYCN*-amplified NB cells than the related chalcone, ISLQ. The reasons for these different effects of similar chalcones require further investigation. However, it is known that the number of hydroxyl groups and their positioning can regulate the ability of chalcones to uncouple the mitochondria by collapsing the mitochondrial membrane [38], suggesting that structural differences between chalcones may account for the differences in the magnitude of their cytotoxic effects on NB cells. Another chalcone, cardamonin, has been found to selectively induce death of human melanoma cells, and not on healthy human melanocytes or dermal fibroblasts [57]. This is consistent with the selectivity cytotoxicity of low doses of 4HC on *MYCN*-amplified NB but not on cultured midbrain neurons, found here in our study. Collectively, these data support the growing body of evidence that chalcone molecules can induce time- and concentration-dependent cytotoxic effects on NB cells.

A substantial issue facing cancer treatments, particularly high-risk metastatic cancers, is resistance to apoptosis. This mechanism has been reported to occur in *MYCN*-amplified tumours, and it confers cell resistance to apoptosis induced by the tumour necrosis factor-related apoptosis-inducing ligand system [58]. The genetic profiles of both of the *MYCN*-amplified cell lines used in the present study may promote cell proliferation rather than differentiation or death via apoptosis [59, 60]. *MYCN* amplification has been also shown to be associated with enriched glutaminolysis [61]. Glutamine is a critical amino acid for many fundamental functions of cancer cells, including synthesis of metabolites that maintain mitochondrial metabolism and generation of antioxidants, such as GSH, to control ROS levels [61]. In cancer cells, the constant proliferation is a highly metabolic process that leads to an enormous accumulation of ROS. Therefore, as an adaptive response, cancer cells harbour elevated levels of ROS-scavenging molecules such as GSH [20]. *MYCN*-amplified NB have been shown to have a twofold enrichment of GSH levels in comparison to cells without *MYCN* amplification (SH-SY5Y) [18]. Moreover, glutaminolysis has been reported to result in excessive amounts of ROS in *MYCN*-amplified NB cells, rendering them even more sensitive to ROS augmentation and increasing their sensitivity to treatment with pro-oxidants such as dimethyl fumarate [61]. These findings are consistent with those of our study, where there was a higher sensitivity to 4HC of *MYCN*-amplified cells, compared to non-*MYCN*-amplified cells. That is, treatment with 5 *μ*M 4HC for 24 h was significantly toxic to IMR-32 and SK-N-BE (2) cells, while 4HC was only toxic at 25 *μ*M for SH-SY5Y and HEK293t cells after the same treatment time.

To confirm whether 4HC-induced cell death targets the delicate balance between extensive GSH production and high levels of ROS in SK-N-BE (2) and IMR-32 cells, we measured GSH levels in 4HC-treated cells, using the GSH recycling assay. We found that treatment of IMR-32 and SK-N-BE (2) with 25-50 *μ*M 4HC caused severe depletion of cellular levels of the antioxidant GSH after 4 h. This was consistent with the findings of a previous study, which found that thirteen distinct hydroxychalcones induced apoptosis in B16-F10 melanoma cells via GSH and ATP depletion [39]. We used CellRox fluorogenic probe to measure oxidative stress in live SK-N-BE (2) cells to determine the level of ROS at various treatment times and concentrations of 4HC. Treatment with 4HC induced an elevation in ROS in a dose- and time-dependent manner. This confirms that 4HC cell toxicity is caused by GSH depletion that is combined with an elevation of ROS levels. Targeting of GSH is a promising approach in the anticancer effect of 4HC, given the role of GSH in promoting tumour progression and metastasis.

We then explored the potential role of the mitochondria in 4HC-induced cell death. Cancer cells have been previously reported to rely on glycolysis, due to their impaired mitochondria [62]. However, other studies have shown that the mitochondria of some cancer cells are functional and not solely dependent on glycolysis for energy requirement, but that they also derive energy from mitochondrial respiration [63, 64]. For example, an isomer of 4-HC has been reported to induce mitochondrial dysfunctional, depletion of intracellular ATP levels, and increases in ROS, in SH-SY5Y cells [46]. In the current study, we detected significant impairment of cellular OCR in 4HC-treated *MYCN*-amplified NB cells, including decreases in ATP-linked respiration, proton leak respiration, and the maximal rate of the electron transport chain, which is reflective of uncoupling. In contrast, nonmitochondrial respiration was not affected by 4HC treatment. Mitochondria are known to maintain a precise balance between bioenergetic and biosynthetic molecular pathways, under normal conditions. In cancer cells, a mismatch in this balance promotes increased ROS generation, favouring metabolic flexibility and mitigating oxidative stress via additional protective mechanisms, such as upregulation of uncoupling proteins [65]. We found that 4HC treatment induced mitochondrial dysfunction in SK-N-BE (2) and IMR-32 cells, by interrupting respiration at several stages. Targeting mitochondrial function in cancer cells has been recently reported as a novel antitumour activity that may counteract drug resistance, inhibit cell proliferation, or decrease cancer metastasis [66–69].

To further investigate the mechanisms involved in 4HC-induced cell death and to confirm the pro-oxidant effect of 4HC, we used IM-54, a small molecule inhibitor of oxidative stress-induced cell death. Pretreatment with IM-54 fully prevented 4HC-induced cell death. Pretreatment with NAC, the precursor of the antioxidant glutathione, also protected cells from 4HC cytotoxicity; this is likely due to compensation of 4HC-induced depletion of GSH [70]. Moreover, pretreatment with Mito-TEMPO, a mitochondria-specific superoxide scavenger, significantly protected cells from 4HC-induced cytotoxicity. All of these findings confirm that 4HC treatment leads to ROS-induced cell death in *MYCN*-amplified NB cells. This is consistent with our previous study showing that another chalcone, ISLQ, induced cell death in *MYCN*-amplified NB cells through the elevation of ROS levels, which was fully prevented by pretreatment with NAC [40]. This is an important finding, as the ability of ROS to chemosensitize cancer cells depends on the basal ROS levels in these cells. ROS levels could represent a double-edged sword, since their activation below a specific threshold promotes cell survival but activation above this threshold can lead to cell death. This may be the reason for the limited success of antioxidant therapies in clinical trials [71] and may support the use of pro-oxidants, including chalcones, in approaches to selectively target malignant cells by exploiting their abnormal levels of ROS [72]. Moreover, selectively targeting the delicate balance of ROS levels in cancer cells is emerging as a potential new strategy [73]. For instance, a novel chalcone derivative, S17, has been found recently to induce apoptosis through ROS-dependent upregulation of DR5 in MGC803 gastric cancer cells; similar to our findings, this death was completely prevented by NAC pretreatment [74].

In this study, we also found that the cytotoxic effects of 4HC increased when this agent was applied in combination with doxorubicin or cisplatin, two of the most commonly used drugs in chemotherapeutic regimens in high-risk NB treatment [75–77]. This is important since high-dose and multiagent chemotherapy regimens can lead to significant side effects. For example, the main dose-limiting side effect of treatment with anthracyclines such as doxorubicin is cardiotoxicity, leading to heart failure in the most severe cases [78]. One in every ten children treated with a cumulative anthracycline dose of 300 mg/m^2^ body surface area or more eventually develops anthracycline-induced clinical heart failure [79]. Doxorubicin has been reported to lead to cardiomyopathy, resulting in a form of congestive heart failure that is usually refractory to common treatment [80]. Moreover, cisplatin has dose-dependent side effects including nephrotoxicity, ototoxicity, and neurotoxicity [81]. For this reason, the use of adjunct agents that allow the application of lower doses of doxorubicin and cisplatin may mitigate against the most severe long-term side effects. Our findings show that 4HC is worthy of further study in this regard.

In addition, GSH has been reported to regulate drug resistance mechanism to cisplatin chemotherapy [82] and doxorubicin [83]. Therefore, cotreatment with 4HC could overcome this problem in chemoresistant NB paediatric cancer. Agents which can modify GSH metabolism, leading to its depletion, could possibly be used to regulate the cellular response to various anticancer agents [84, 85]. For example, GSH depletion has been reported to increase glioma cancer cell sensitivity to both cisplatin and temozolomide [86] and leukemia cell sensitivity to doxorubicin and etoposide [87]. Therefore, depletion of GSH induced by 4HC could enhance NB cell sensitivity to treatment with anticancer agents. Moreover, the presence of a high uncoupling rate in malignant tumours, rather than an indicator of oxidative stress, could be an antiapoptotic mechanism that results in increased survival and chemoresistance ability [88]. Therefore, the use of 4HC as a supplement or cotreatment could enhance NB cells' responses to mainstream chemotherapy agents via several methods.

In this study, we show that 4HC is a dietary compound that has a higher selective cytotoxicity on *MYCN*-amplified NB cells than on other cell types. This cytotoxic effect involved depletion of GSH and elevation of ROS levels. Application of 4HC in combination with well-known chemotherapy agents led to a greater toxicity on SK-N-BE (2) and IMR-32 in relatively low doses; this combined application could potentially be used to overcome drug resistance issues associated with the use of high doses of chemotherapy agents. Therefore, the use of 4HC alone, or in combination with mainstream antitumour drugs, may have potential for NB treatment, in particular if it allowed the use of lower doses of chemotherapeutic agents, reducing the incidence of toxic long-term side effects.

## 5. Conclusion

*MYCN* amplification occurs in ∼30% of high-risk NB cases, influences drug resistance [89]. Therefore, the search for novel drugs with fewer side effects and/or with greater therapeutic efficiency is a key priority for NB therapy, due to the rapid development of resistance against chemotherapeutic drugs and their undesirable side effects [90]. This study shows that 4HC exerts potent cytotoxic effects on *MYCN*-amplified NB cells and that it can be combined with clinically approved anticancer drugs, doxorubicin or cisplatin, to potentiate NB cell death. In summary, this provides important proof-of-principle that rationalises further study of 4HC as a therapeutic agent of interest for NB.

## Data Availability

The data used to support the findings of this study are available from the corresponding author upon request.

## Abbreviations

4HC: 4-Hydroxychalcone
GSH: Glutathione
NAC: N-acetyl-L-cysteine
MeOH: Methanol
NB: Neuroblastoma
MTT: 3-(4,5-Dimethylthiazol-2-yl)-2,5-diphenyltetrazolium bromide
ROS: Reactive oxygen species.

## Supplementary Materials

Supplementary Materials In this study, supplementary materials consist of four figures. Supplementary Figure 1: treatment with vehicle control does not affect cell viability. Cell viability as measured using MTT assay on (A) SK-N-BE (2), (B) IMR-32, (C) SH-SY5Y, and (D) HEK293t cell lines, treated with the indicated concentrations of vehicle (MeOH) control for 24 h. All data are mean ± SEM; ANOVA with post hoc Tukey's test. Supplementary Figure 2: NB cells are more sensitive to 4HC than to ISLQ. (A, B) Cell viability as measured using MTT assay and (C) representative photomicrographs of SK-N-BE (2) and IMR-32 cell lines, as indicated, treated with 10 *μ*M of ISLQ or 4HC for 24 h. The ISLQ data have been previously published [41] All data are mean ± SEM; *n* = 3 independent experiments. ∗*p* < 0.05 and ∗∗∗*p* < 0.001 versus control; ANOVA with post hoc Tukey's test. Supplementary Figure 3: 4HC-induced oxidative stress is prevented by IM-54. (A) Representative photomicrographs and (B) relative CellRox fluorescence intensity in SK-N-BE (2) cells treated with 25 *μ*M of 4HC for 24 h with or without pretreatment with 3 *μ*M IM-54. Arrows show elevated ROS. Scale bar = 50 *μ*m. All data are mean ± SEM; *n* = 3 independent experiments. ∗∗*p* < 0.01 and ∗∗∗*p* < 0.001 versus control or as indicated; ANOVA with Tukey's post hoc test. Supplementary Figure 4: effects of increasing Dox concentrations on cell viability. (A, B) Cell viability as measured using MTT assay on (A) SK-N-BE (2), (B) IMR-32, (C) SH-SY5Y, and (D) HEK293t cell lines, treated with the indicated concentrations of doxorubicin (Dox) for 24 h. All data are mean ± SEM; *n* = 3 independent experiments. ∗*p* < 0.05 and ∗∗∗*p* < 0.001 versus control; ANOVA with post hoc Tukey's test.
