# Supplementary material for: 4-Hydroxychalcone Induces Cell Death via Oxidative Stress in MYCN-Amplified Human Neuroblastoma Cells
Source: Oxid Med Cell Longev. 2019 Dec 5;2019:1670759. doi: 10.1155/2019/1670759 (PMC6915131; doi:10.1155/2019/1670759)
Supplement: Supplementary Materials — In this study, supplementary materials consist of four figures. Supplementary Figure 1: treatment with vehicle control does not affect cell viability. Cell viability as measured using MTT assay on (A) SK-N-BE (2), (B) IMR-32, (C) SH-SY5Y, and (D) HEK293t cell lines, treated with the indicated concentrations of vehicle (MeOH) control for 24 h. All data are mean ± SEM; ANOVA with post hoc Tukey's test. Supplementary Figure 2: NB cells are more sensitive to 4HC than to ISLQ. (A, B) Cell viability as measured using MTT assay and (C) representative photomicrographs of SK-N-BE (2) and IMR-32 cell lines, as indicated, treated with 10 μM of ISLQ or 4HC for 24 h. The ISLQ data have been previously published [41] All data are mean ± SEM; n = 3 independent experiments. ∗p < 0.05 and ∗∗∗p < 0.001 versus control; ANOVA with post hoc Tukey's test. Supplementary Figure 3: 4HC-induced oxidative stress is prevented by IM-54. (A) Representative photomicrographs and (B) relative CellRox fluorescence intensity in SK-N-BE (2) cells treated with 25 μM of 4HC for 24 h with or without pretreatment with 3 μM IM-54. Arrows show elevated ROS. Scale bar = 50 μm. All data are mean ± SEM; n = 3 independent experiments. ∗∗p < 0.01 and ∗∗∗p < 0.001 versus control or as indicated; ANOVA with Tukey's post hoc test. Supplementary Figure 4: effects of increasing Dox concentrations on cell viability. (A, B) Cell viability as measured using MTT assay on (A) SK-N-BE (2), (B) IMR-32, (C) SH-SY5Y, and (D) HEK293t cell lines, treated with the indicated concentrations of doxorubicin (Dox) for 24 h. All data are mean ± SEM; n = 3 independent experiments. ∗p < 0.05 and ∗∗∗p < 0.001 versus control; ANOVA with post hoc Tukey's test. [file 1670759.f1.docx]

**Supplementary Materials**

In this study, supplementary materials consist of four figures.


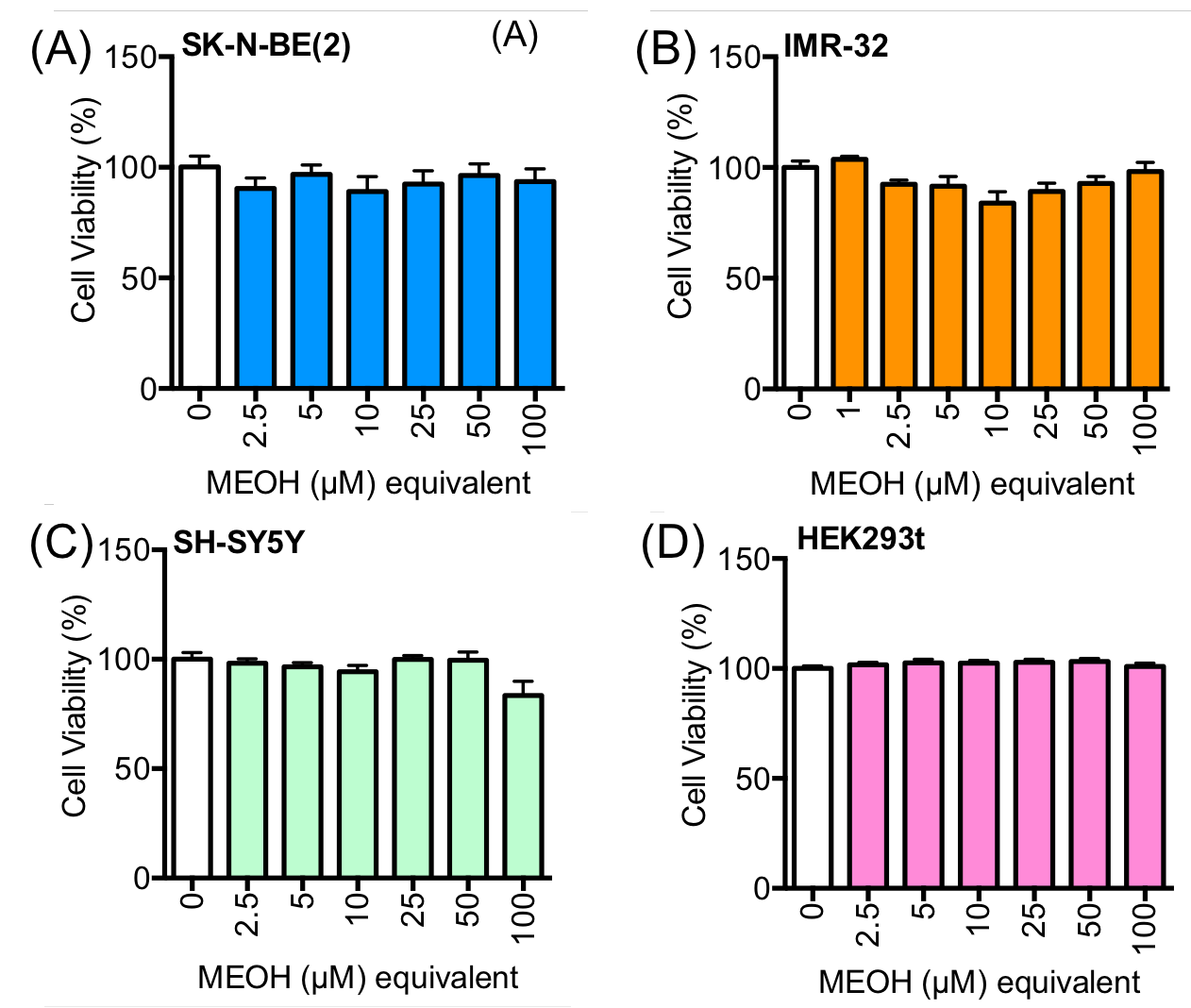


**Supplementary Figure 1: Treatment with vehicle control does not affect cell viability.**

Cell viability as measured using MTT assay on (A) SK-N-BE(2), (B) IMR-32, (C) SH-SY5Y and (D) HEK293t cell lines, treated with the indicated concentrations of vehicle (MeOH) control for 24 h. All data are mean ± sem; ANOVA with *post-hoc* Tukey’s test.


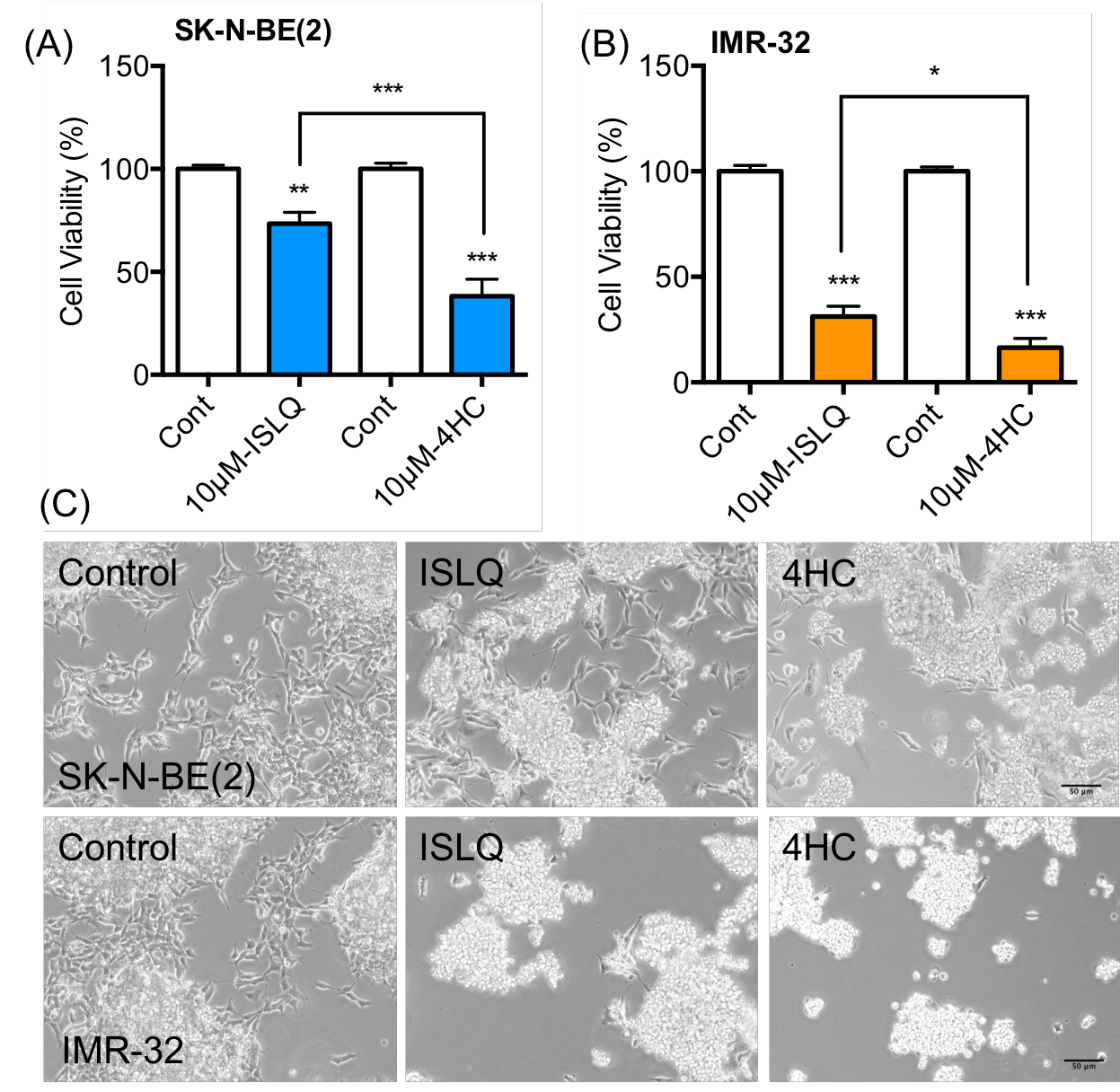


**Supplementary Figure 2: NB cells are more sensitive to 4HC than to ISLQ.**

(A, B) Cell viability as measured using MTT assay and (C) representative photomicrographs of SK-N-BE(2) and IMR-32 cell lines, as indicated, treated with 10µM of ISLQ or 4HC for 24 h. The ISLQ data have been previously published [41] All data are mean ± sem; *n* =3 independent experiments. *p < 0.05, ***p < 0.001 versus control; ANOVA with *post-hoc* Tukey’s test.


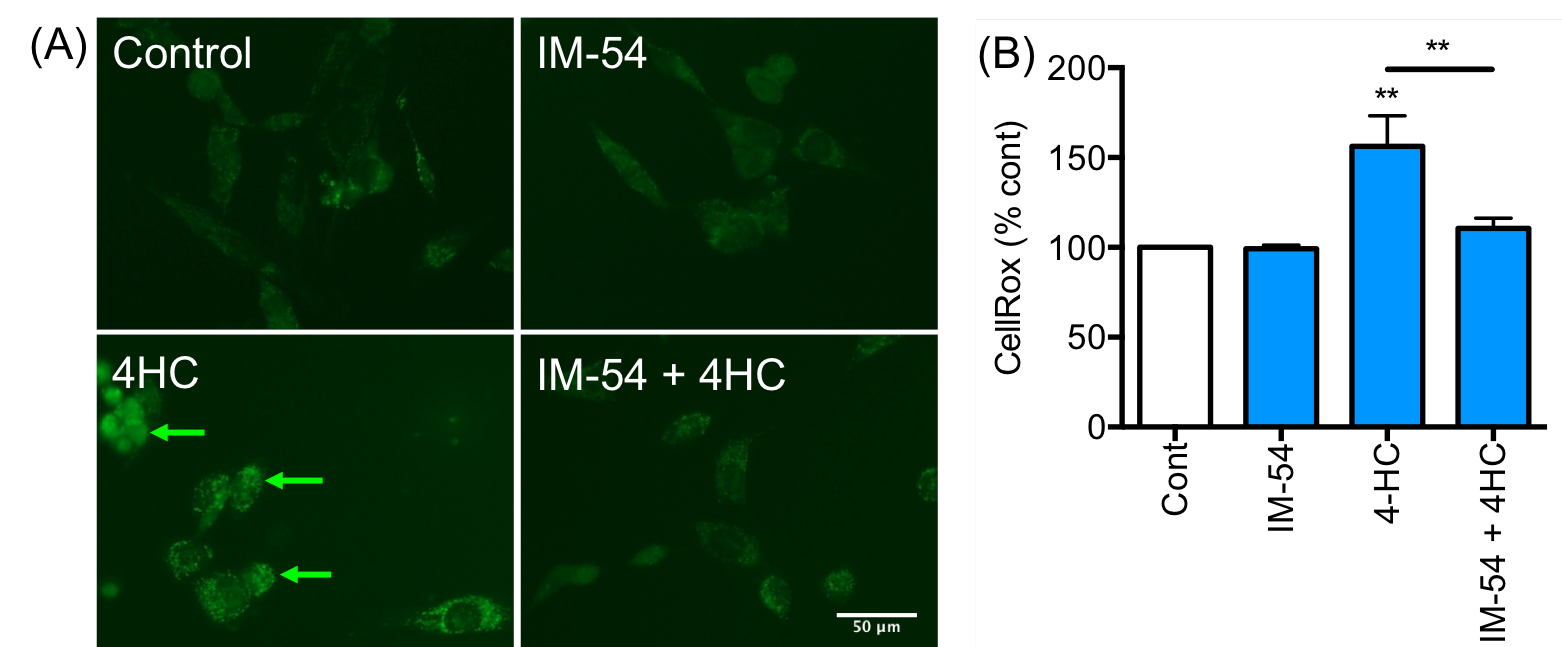


**Supplementary Figure 3: 4HC-induced oxidative stress is prevented by IM-54.**

(A) Representative photomicrographs of and (B) relative CellRox fluorescence intensity in SK-N-BE(2) cells treated with 25 µM of 4HC for 24 h with or without pretreatment with 3 μM IM-54. Arrows show elevated ROS. Scale bar = 50 µm. All data are mean ± sem; *n*=3 independent experiments. ** p < 0.01, ***p < 0.001 vs. Control or as indicated; ANOVA with Tukey’s post-hoc test.


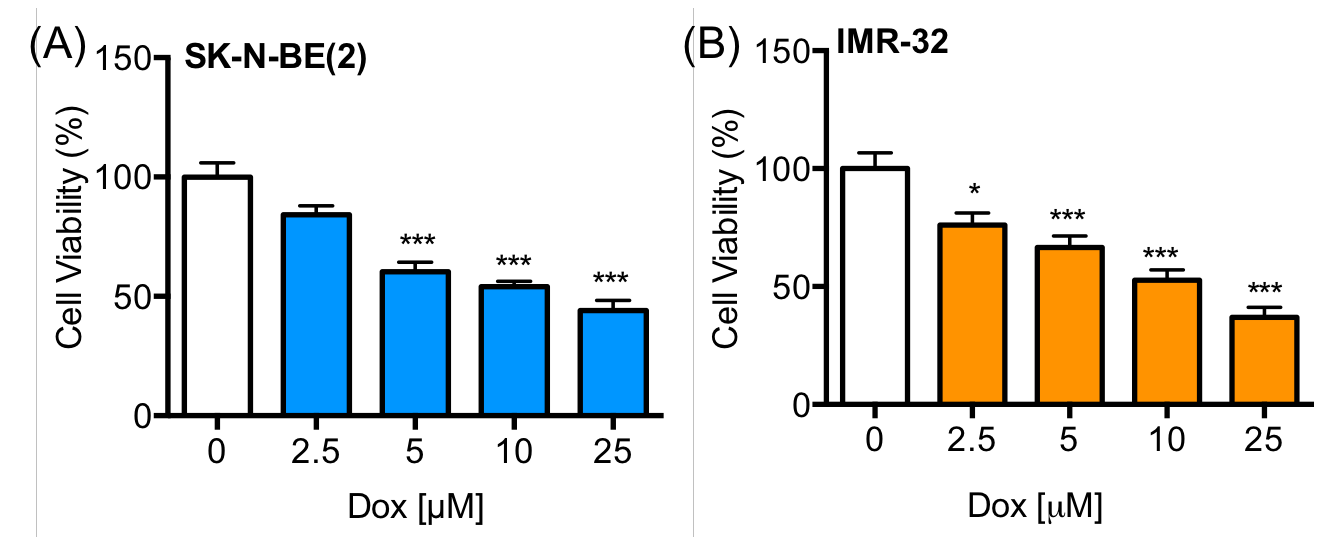


**Supplementary Figure 4: Effects of increasing Dox concentrations on cell viability.**

(A, B) Cell viability as measured using MTT assay on (A) SK-N-BE(2), (B) IMR-32, (C) SH-SY5Y and (D) HEK293t cell lines, treated with the indicated concentrations of doxorubicin (Dox) for 24 h. All data are mean ± sem; *n* =3 independent experiments. *p < 0.05, ***p < 0.001 versus control; ANOVA with *post-hoc* Tukey’s test.
